# Characterization of interactions within the Igα/Igβ transmembrane domains of the human B-cell receptor provides insights into receptor assembly

**DOI:** 10.1016/j.jbc.2022.101843

**Published:** 2022-03-18

**Authors:** Christine Lockey, Hannah Young, Jessica Brown, Ann M. Dixon

**Affiliations:** Department of Chemistry, University of Warwick, Coventry, UK

**Keywords:** B-cell receptor (BCR), CD79, transmembrane domain, protein–protein interaction, GALLEX, lipid raft, circular dichroism (CD), protein cross-linking, β-gal, β-galactosidase, AP2, adaptor protein 2, BCR, B-cell receptor, CHI, CNS searching of helix interactions, DPC, dodecylphosphocholine, ER, endoplasmic reticulum, GpA, glycophorin A, IMAC, immobilized metal affinity chromatography, ITAM, immunoreceptor tyrosine-based activation motif, mIg, membrane-bound immunoglobulin, MW, molecular weight, TMD, transmembrane domain

## Abstract

The B-cell receptor (BCR), a complex comprised of a membrane-associated immunoglobulin and the Igα/β heterodimer, is one of the most important immune receptors in humans and controls B-cell development, activity, selection, and death. BCR signaling plays key roles in autoimmune diseases and lymphoproliferative disorders, yet, despite the clinical significance of this protein complex, key regions (*i.e.*, the transmembrane domains) have yet to be structurally characterized. The mechanism for BCR signaling also remains unclear and has been variously described by the mutually exclusive cross-linking and dissociation activation models. Common to these models is the significance of local plasma membrane composition, which implies that interactions between BCR transmembrane domains (TMDs) play a role in receptor functionality. Here we used an *in vivo* assay of TMD oligomerization called GALLEX alongside spectroscopic and computational methods to characterize the structures and interactions of human Igα and Igβ TMDs in detergent micelles and natural membranes. We observed weak self-association of the Igβ TMD and strong self-association of the Igα TMD, which scanning mutagenesis revealed was entirely stabilized by an E–X_10_–P motif. We also demonstrated strong heterotypic interactions between the Igα and Igβ TMDs both *in vitro* and *in vivo*, which scanning mutagenesis and computational models suggest is multiconfigurational but can accommodate distinct interaction sites for self-interactions and heterotypic interactions of the Igα TMD. Taken together, these results demonstrate that the TMDs of the human BCR are sites of strong protein–protein interactions that may direct BCR assembly, endoplasmic reticulum retention, and immune signaling.

The B-cell receptor (BCR) is composed of an antigen-binding subunit, the membrane-bound immunoglobulin (mIg), and a signal transduction subunit, the Igα/Igβ (or CD79a/b) heterodimer. The BCR is found on the surface of B cells and is responsible for activating naïve and memory B cells upon binding of intact pathogenic antigens to highly specific binding sites on the mIg. Antigen binding triggers intracellular signaling *via* Igα/Igβ, leading to antibody production and endocytosis of the antigen–BCR complex for subsequent presentation to T cells *via* the Class II major histocompatibility complex. The activation of B cells in this way is essential to the humoral immune response. When B-cell activation fails, this can manifest as a tolerance to foreign antigens and a failure to respond to vaccinations (1). Conversely, aberrant BCR signaling leads to allergy (2), autoimmune diseases (3), leukemias (4, 5), and lymphomas (6). Thus, the BCR represents a therapeutic target of great clinical potential.

Given the biological importance of the BCR, a structural understanding of this receptor is of great medical interest. Indeed, many aspects of BCR structure and assembly are well understood and are summarized in Figure 1*A*. The mIg molecule is a symmetrical, disulfide-linked homodimer consisting of two heavy chains which span the membrane and two light chains that complete the antigen-binding site. It has no intracellular component; therefore, intracellular signaling is mediated entirely by the Igα/Igβ heterodimer. Igα and Igβ are both type I transmembrane proteins comprised of an extracellular Ig-like domain, a transmembrane domain (TMD), and a cytoplasmic domain. The cytoplasmic domains of both Igα and Igβ contain an immunoreceptor tyrosine-based activation motif (ITAM) and mediate Ca^2+^ mobilization; however, only Igα mediates protein tyrosine kinase activation and interleukin-2 expression (*e.g.*, in B-cell lymphoma) (7). Igα and Igβ are coexpressed in the endoplasmic reticulum (ER) (8) where they form a disulfide-linked Igα/Igβ heterodimer that then binds to mIg. While the reported stoichiometry of components in the BCR has varied from a 1:2 stoichiometry, in which two copies of the Igα/Igβ heterodimer bind to an mIg homodimer (9), to the more recent 1:1 model (*i.e.*, one copy of the Igα/Igβ heterodimer bound to each mIg homodimer) (10, 11, 12), the stoichiometry of the Igα/Igβ heterodimer remains unquestioned. It is this interaction that represents the fundamental first step in formation of intact BCR competent for transport to the cell surface (13, 14). Subsequent to antigen binding, the ligand–BCR complex is internalized by the B cell through a process of endocytosis mediated by the Igα and Igβ cytoplasmic domains (15). Mutagenesis studies have identified that a crucial Tyr motif in the cytoplasmic domain of Igβ is required for adaptor protein 2 (AP2)–mediated endocytosis but that AP2–BCR interactions initially occur *via* the cytoplasmic domain of Igα (16). Thus, the very last steps in BCR activity are also directed by the Igαβ heterodimer, and a molecular description of this species is of great value to our understanding of the receptor as a whole.

**Figure 1:**
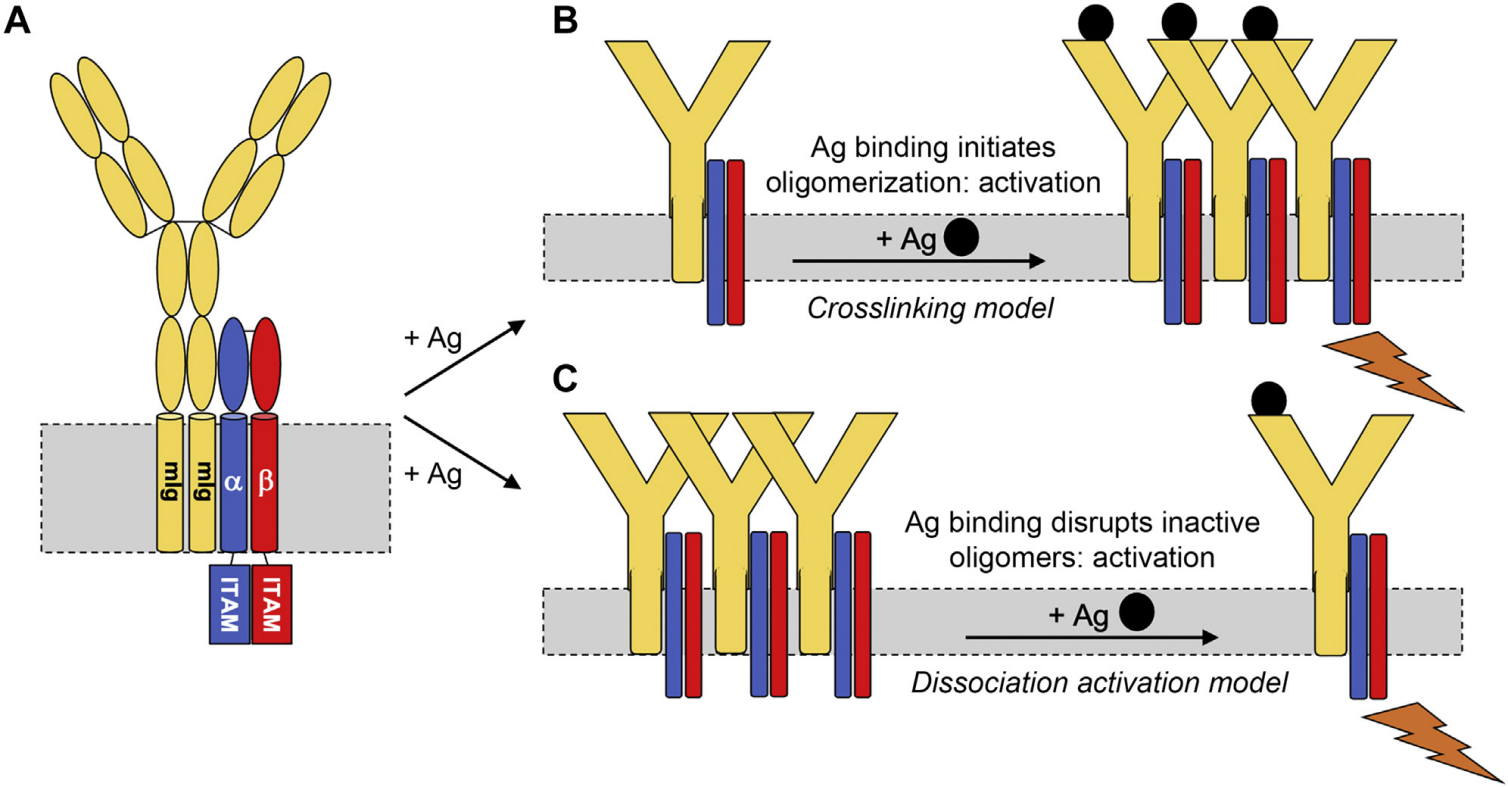
**Structural models of the B-cell receptor.***A*, schematic of the human B-cell receptor (BCR) complex in the plasma membrane, composed of the membrane-spanning immunoglobulin (mIg) bound to an Igα–Igβ heterodimer. Igα and Igβ are type I membrane proteins containing an extracellular Ig-like domain, a transmembrane domain, and a cytoplasmic domain with an immunoreceptor tyrosine-based activation motif (ITAM) that mediates Ca^2+^ mobilization. The two predominant models for BCR activation are (*B*) the cross-linking model, in which antigen (Ag) binding initiates BCR oligomerization and signaling, and (*C*) the dissociation activation model, in which antigen binding dissociates inactive BCR oligomers and leads to signaling.

Here, we wished to characterize the structure and interactions of the TMDs of both Igα and Igβ in natural membranes and membrane mimetic environments. It is well known that the Igα/Igβ heterodimer is stabilized by a disulfide bond between the extracellular Ig-like domains of each protein (8), and the Igβ Ig-like domain has been structurally characterized in great detail (17). However, while the Ig-like domains of Igα and Igβ are essential for BCR assembly (18, 19, 20) and membrane translocation (21), they are dispensable to BCR signaling (22). Intrareceptor signaling is believed to be localized to interactions between the TMDs, where mIg, Igα, and Igβ share space in the membrane bilayer. Supporting this hypothesis is the report that residues within the TMDs of Igα and Igβ, specifically an E/Q-X_10_-P motif, direct ER retention and association with the mIg homodimer (12). Similarly, mutation of residues in the membrane-bound IgM and membrane-bound IgD TMDs disrupts interactions with the Igα/Igβ heterodimer (10, 23, 24, 25, 26). Surprisingly, little study has been made of the Igα/Igβ TMD interactions. Computational analyses identified juxtamembrane residues in Igα and Igβ which stabilized a putative Igαβ heterodimer, with little contribution from residues within the TMDs (27); however, this investigation did not extend to the mechanism by which signaling is propagated through the BCR, and the role of Igα/Igβ TMD residues in this context is unclear.

The issue is further complicated by the fact that there exists a dynamic relationship between the BCR and the plasma membrane. BCRs have been shown to cluster in the membrane (28), segregated by mIg type, and form nanoclusters on the order of 100 nm in diameter. Upon activation, these nanoclusters are altered, becoming smaller and more dispersed (29). The composition of the local membrane varies during BCR activation; the BCR in resting B cells is excluded from cholesterol-enriched lipid rafts but becomes associated with rafts immediately following antigen binding (30, 31, 32, 33). BCR isolated from lipid rafts is phosphorylated (30, 31, 32), suggesting rafts are the site of BCR ITAM phosphorylation. The driving force of interactions between the BCR and membrane lipids is not understood; however, these interactions must involve the TMDs of the BCR.

BCR signaling has variously been described using two competing models. In the cross-linking model (Fig. 1*B*), BCRs form higher-order oligomers upon antigen binding, and this oligomerization initiates receptor signaling. Conversely, in the dissociation activation model (Fig. 1*C*), inactive BCRs form autoinhibited oligomers at the cell surface which are disrupted by antigen binding, increasing the accessibility of Igα/β ITAMs to cytosolic kinases to permit signaling (14). In both models, the distinction between monomeric and oligomeric BCRs is key to signal propagation. Indeed, the assembly of higher-order receptor clusters has been reported to drive signal transduction in several other proteins (34). A holistic understanding of BCR function must include the mechanisms that guide receptor clustering, and we suggest that the TMDs could contribute strong sites of interaction.

Despite this building evidence, no experimental data have yet been reported documenting the structure and interactions of these TMDs in isolation. Characterization of these interactions would bridge an existing gap in our understanding of assembly, signal transduction, and clustering of the BCR. We have therefore used a combination of biophysical, biochemical, and computational methods to characterize the Igα and Igβ TMDs in micelles and natural membranes. We used circular dichroism (CD) spectroscopy and chemical cross-linking to study the *in vitro* folding and self-assembly of synthetic peptides corresponding to the Igα and Igβ TMDs *in vitro* in detergent micelles. These interactions were further investigated in a natural membrane using the GALLEX assay (35), and site-directed mutagenesis was used to identify the molecular determinants of these interactions. We report for the first time the weak self-association of the Igβ TMD and the strong self-association of the Igα TMD, which scanning mutagenesis revealed was entirely stabilized by an E–X_10_–P motif previously reported to direct ER retention and interactions between Igα and mIg (12). We also describe strong heterotypic interactions between the Igα and Igβ TMDs both *in vitro* and *in vivo*, which scanning mutagenesis and computational models suggest is less well defined and potentially multiconfigurational. From these models, it is clear that the Igα TMD can accommodate separate interaction sites for self-interactions and heterotypic interactions of the Igα TMD that could occur concurrently and contribute to the assembly, localization, and/or clustering of BCRs observed in the B-cell plasma membrane.

## Results

### Secondary structure and oligomeric state of human Igα and Igβ transmembrane domains in detergent micelles

The sequences of the Igα and Igβ TMDs are highly conserved across species (12). To determine the secondary structure of the human Igα and Igβ TMDs (which have not been reported to date), peptides derived from the TMDs of both proteins were prepared. Peptides contained putative TMD residues (12) plus two to four juxtamembrane residues at each terminus to aid solubility. A hexahistidine tag was added to the N-terminus of the Igβ peptide to facilitate affinity chromatography, and a non-native Trp was added to the N-termini of the Igα and Igβ peptides for concentration determination and selective fluorescence detection (discussed later). All peptide sequences are shown in Table 1.

**Table 1:** The sequences of Igα and Igβ transmembrane domains (TMDs) from *Homo sapiens*; a series of 18-residue truncations generated for the *in vivo* GALLEX assay; and the sequences of three synthetic peptides designed to mimic Igα and Igβ for *in vitro* study

Igα	Amino acid sequence
*Native TMD sequence*	RIITAEGIILLFCAVVPGTLLLFR
*GALLEX N-term R*_*143*_*-G*_*160*_	RIITAEGIILLFCAVVPG
*GALLEX Core T*_*146*_*-L*_*163*_	TAEGIILLFCAVVPGTLL
*GALLEX C-term G*_*149*_*-R*_*166*_	GIILLFCAVVPGTLLLFR
*Igα TMD peptide*	WTKNRIITAEGIILLFCAVVPGTLLLFRKR


Igβ	Amino acid sequence
*Native TMD sequence*	DGIIMIQTLLIILFIIVPIFLLLD
*GALLEX N-term D*_*158*_*-P*_*175*_	DGIIMIQTLLIILFIIVP
*GALLEX Core I*_*161*_*-L*_*178*_	IMIQTLLIILFIIVPIFL
*GALLEX C-term Q*_*164*_*-D*_*181*_	QTLLIILFIIVPIFLLLD
*Igβ TMD peptide*	WKDGIIMIQTLLIILFIIVPIFLLLDKDDS
*His*_*6*_*-Igβ TMD peptide*	HHHHHHKDGIIMIQTLLIILFIIVPIFLLLDKDDS

**Figure 2:**
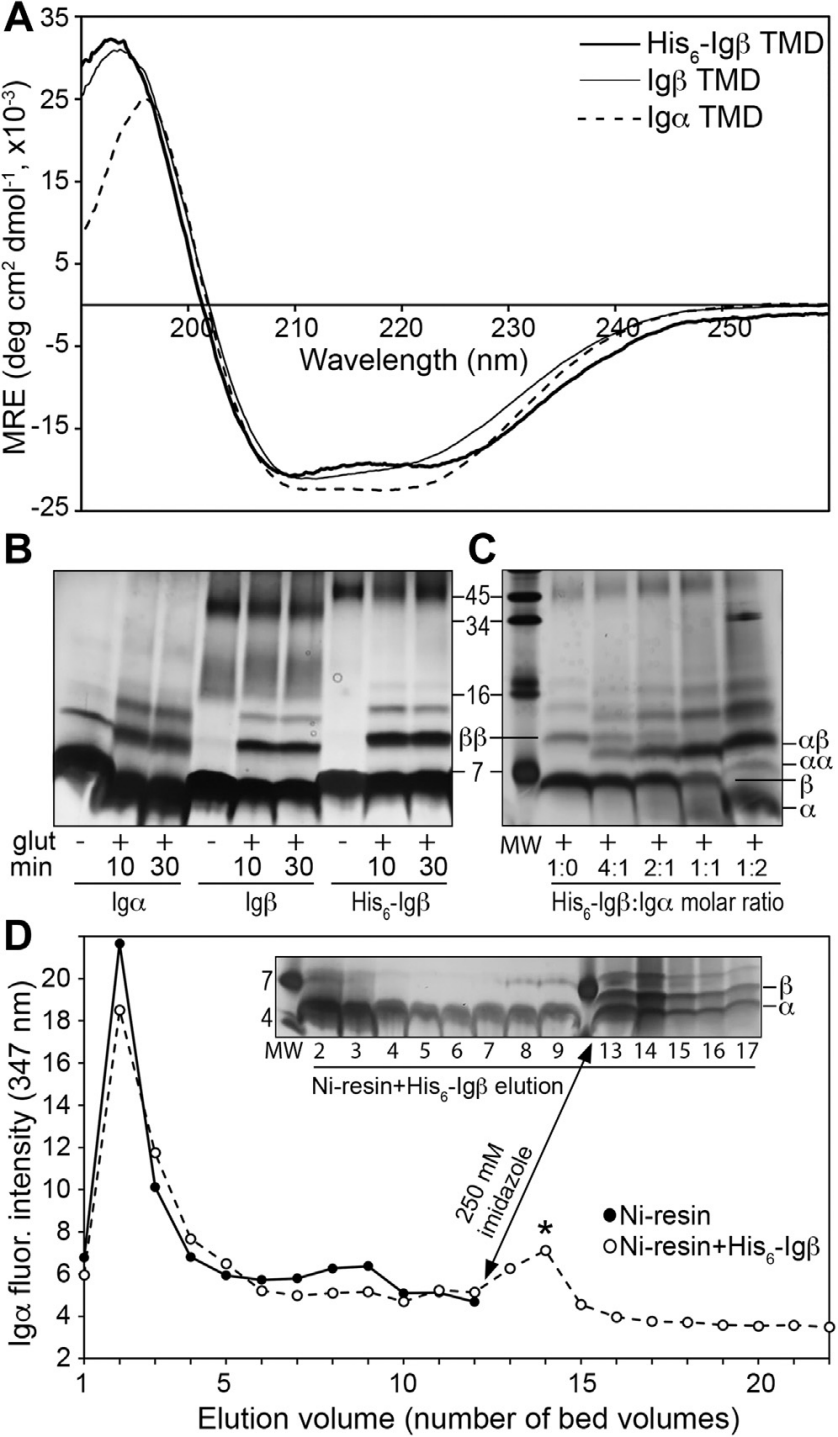
**Secondary structure and interactions of Igα and Igβ TMD peptides *in vitro*.***A*, Circular dichroism spectra of peptides derived from Igα and Igβ TMDs (Table 1) solubilized in 100 mM DPC detergent. All CD spectra, given in units of mean residue ellipticity (MRE), display a characteristically α-helical shape with a maximum around 195 nm and minima at 208 and 222 nm. *B* and *C*, SDS-PAGE analyses of Igα and Igβ TMD peptides in 50 mM DPC micelles in the absence (-) and presence (+) of the chemical cross-linker glutaraldehyde, where cross-linking was carried out for 10 or 30 min before quenching. Peptides were analyzed individually (*B*) as well as in mixtures of varying molar ratio with the His_6_-Igβ peptide concentration held constant (*C*) and were visualized by staining with silver nitrate. Monomeric (α, β) and dimeric (αα, ββ, αβ) species are indicated. Migration was compared to that of a series of standards (MW) whose masses are given in kDa. *D*, Selective fluorescence detection of the Igα TMD peptide *via* its non-native Trp residue in elution fractions from an IMAC column containing Ni-charged Sepharose in the absence and presence of Ni-bound His_6_-Igβ TMD peptide. Addition of imidazole yielded elution of bound Igα TMD peptide (∗). (inset) SDS-PAGE analyses of elution fractions from IMAC column containing Ni-bound His_6_-Igβ TMD peptide, where addition of imidazole yielded elution of both Igα and His_6_-Igβ peptides. DPC, dodecylphosphocholine; IMAC, immobilized metal affinity chromatography; MW, molecular weight; TMD, transmembrane domain.

Peptides were reconstituted into the zwitterionic detergent dodecylphosphocholine (DPC), shown in the past to be highly amenable to structural investigations of transmembrane peptides and proteins (36, 37, 38, 39, 40). Figure 2*A* shows the resulting CD spectra for each peptide solubilized in 25 mM sodium phosphate buffer, pH 7.4, containing 100 mM DPC. All spectra show the characteristic features of an α-helical secondary structure, with negative peaks at 208 and 222 nm and a positive peak near 195 nm. These data are the first (to our knowledge) confirming the helical structure of the Igα and Igβ TMDs experimentally.

SDS-PAGE in combination with chemical cross-linking was used to explore self-assembly of each TMD peptide solubilized in 50 mM DPC (Fig. 2*B*). The Igα TMD peptide (molecular weight [MW] = 3.4 kDa) yielded clear bands corresponding to monomer and dimer species. Cross-linking using the amine-selective cross-linker glutaraldehyde stabilized a further species which could be either a trimer or tetramer. Conversely, the Igβ and His_6_-Igβ TMD peptides (MW = 3.5 and 4.1 kDa, respectively) were predominantly monomeric in the absence of a cross-linker, forming no SDS-stable oligomers. These two peptides also behaved identically upon addition of glutaraldehyde, yielding cross-linker-stabilized dimeric and trimeric/tetrameric species. Likewise, both peptides yielded a higher-order aggregate, which we approximate to be a 10 to 11 mer, which was not impacted by cross-linking. Taken together, these results suggest that while both TMDs can support oligomerization once a stabilizing influence (*i.e.*, glutaraldehyde) is present, self-association of the Igα TMD is more stable than that of the Igβ TMD in isolation.

### Self-association of the human Igα and Igβ transmembrane domains in a natural membrane environment

TMD self-association in a natural membrane bilayer was studied using the GALLEX assay (35). GALLEX is a two-plasmid, LexA-based transcriptional assay linked to β-galactosidase (β-gal) expression, where a TMD of interest is inserted between maltose-binding protein (MalE) and the N-terminal domain (residues 1–87) of LexA. Association of the resulting chimeras (*via* TMD interactions) leads to repression of β-gal expression and thus indicates strong helix–helix interactions in the inner membrane of *Escherichia coli*. This assay can be used to monitor both homo- and hetero-association and was ideal for use in this work. Oligonucleotide primers encoding 18-residue sections of the Igα and Igβ TMDs were cloned into the GALLEX chimera as described in the Experimental procedures. While the native TMDs are predicted to stretch over approximately 24 amino acids, TMD regions of 17 to 18 amino acids in length have been reported as optimal for GALLEX (35, 41). The entire length of each TMD was sampled by our measurements through preparation of three constructs for each protein: an “N-terminal” construct containing the first 18 residues of the TMD, a “core” construct containing the central 18 residues, and a “C-terminal” construct containing the final 18 residues. All sequences screened are summarized in Table 1, where the large degree of sequence overlap (15 residues) from one construct to the next can be seen. GALLEX chimeras were also generated encoding the strongly dimeric TMD of glycophorin A (GpA) (42, 43, 44) as a positive control and its dimerization-compromised point mutant G_83_I (45) as a negative control.

**Figure 3:**
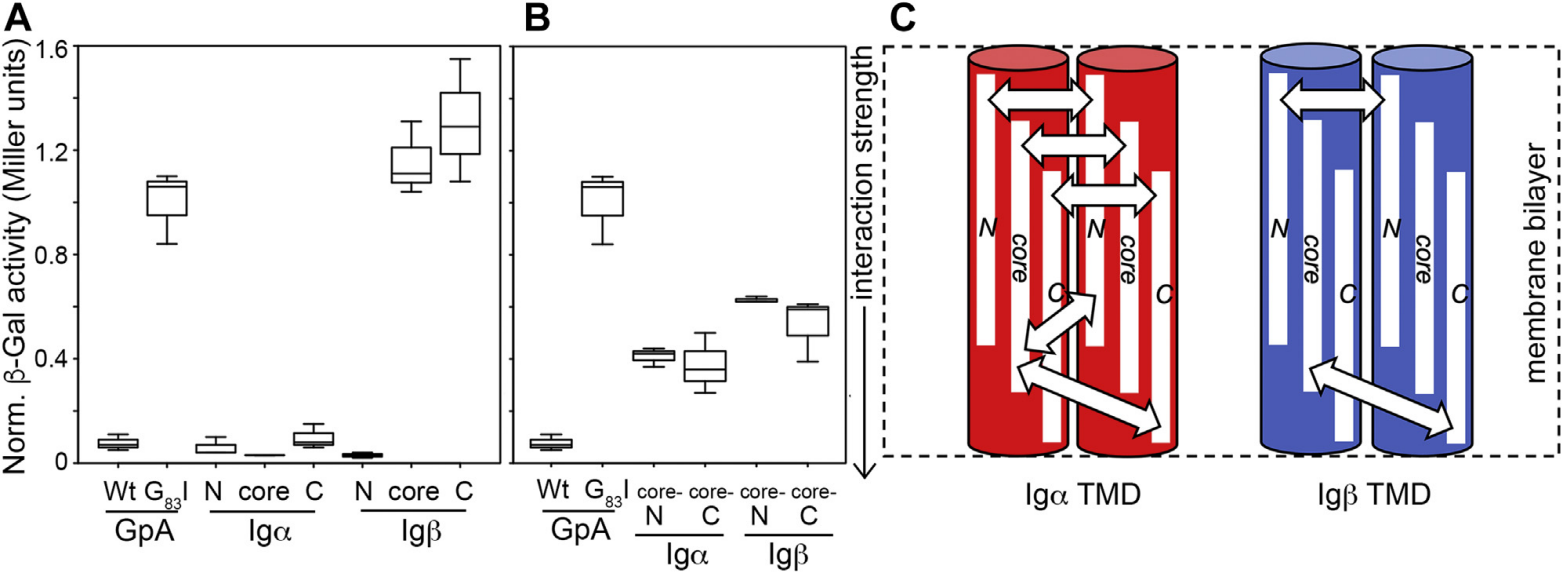
**Self-association of the BCR transmembrane domains (TMDs) in a natural membrane bilayer.***A*, Box plot of homotypic GALLEX data collected for three separate frames (*i.e.*, N-terminal, core, and C-terminal) of the Igα and Igβ TMD sequences (see Table 1), showing the median, interquartile range, and extremes of each dataset. *B*, The heterotypic GALLEX assay was also used to investigate interaction between different regions of the same TMD. For example, interactions between the core region and the N-terminal region of the Igα TMD were studied. All values were compared to a positive control, the TMD of glycophorin A (GpA), and its dimerization-compromised mutant G_83_I, with all data provided in Miller units after normalization to expression level (Fig. S1) and the value obtained from G_83_I. Reported values are derived from three to six biological repeats. *C*, Schematic summary of GALLEX data for the three different frames of the Igα and Igβ TMDs, with double-headed arrows indicating which regions of the TMDs associate strongly. These data suggest that the Igα TMD strongly self-associates along its entire length, while the Igβ TMD shows a more sparse pattern of helix–helix interactions in the membrane. BCR, B-cell receptor.

Figure 3*A* shows box plots of the GALLEX results obtained for homotypic interactions between the N-terminal, core, and C-terminal TMD regions of wildtype human Igα and Igβ. Heterotypic interactions between different regions of the same TMD (*e.g.*, between the core and N-terminal regions of a given TMD) were also investigated using GALLEX, and the results are shown in Figure 3*B*. In all cases, interaction strength was reported relative to the positive (GpA) and negative (G_83_I) controls after normalization to protein expression level (obtained *via* Western blot, see Fig. S1) and the β-Gal activity observed for G_83_I. The GALLEX data are summarized in a schematic shown in Figure 3*C* and clearly indicate that the Igα TMD strongly self-associates along its entire length. Observed interaction strengths for the N-terminal, core, and C-terminal fragments are all comparable to that observed for wildtype GpA. Additionally, the core region of Igα associates with both the N- and C-terminal regions of the same TMD, suggesting the amino acid sequence common to all Igα TMD constructs (specifically GIILLFCAVVPG) may direct this interaction. Conversely, only the N-terminal fragment of Igβ strongly self-associates. Removal of the three N-terminal amino acids (DGI, as in the central core construct) abolishes this interaction, suggesting that the observed interaction is not localized to the TMD but instead resides in the juxtamembrane region of Igβ. Likewise, the C-terminal region of the Igβ TMD yielded interactions that were weaker than that observed for G_83_I. The core region of Igβ does appear to associate weakly with both the N- or C-terminal regions of the same TMD. These results echo those obtained *in vitro* for both TMDs solubilized in detergent micelles (Fig. 2*B*).

### Homo-oligomerization of the Ig*α* transmembrane domain in isolation is mediated by an E–X_10_–P motif

**Figure 4:**
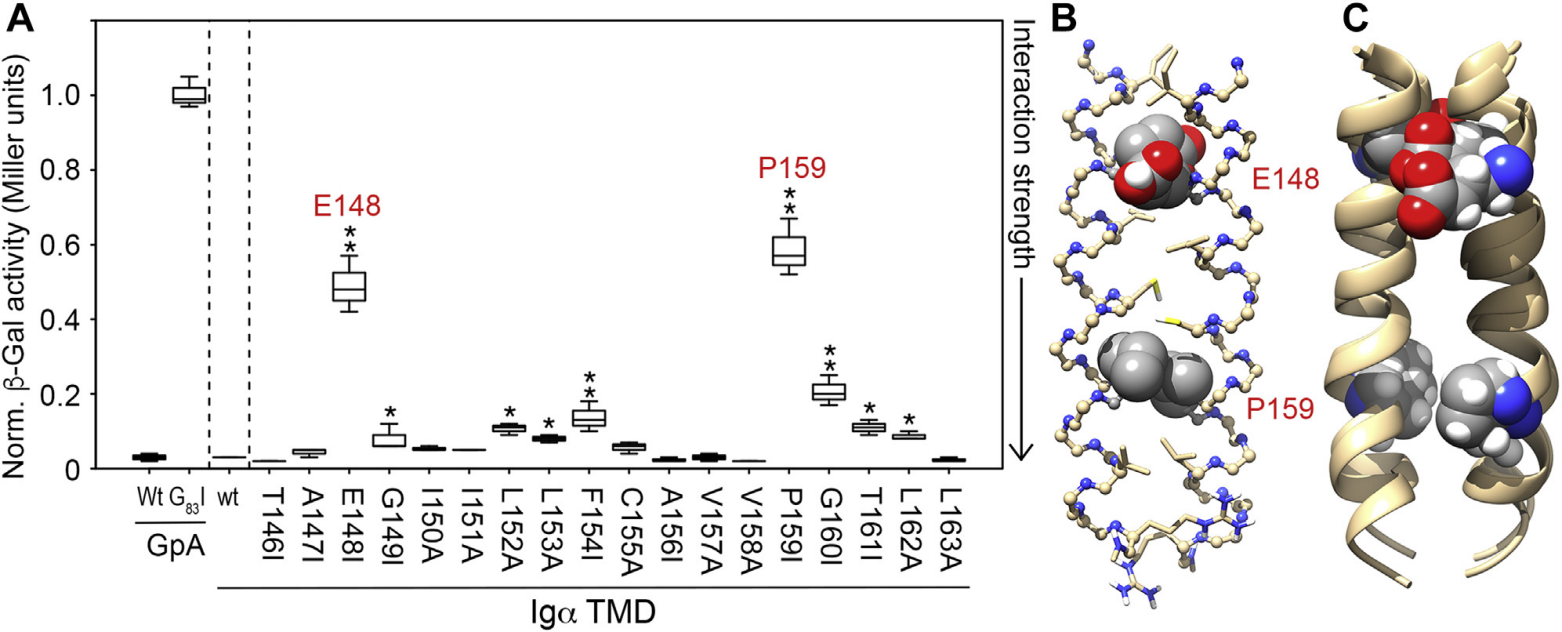
**Identification of E–X**_**10**_**–P interaction site in Igα TMD homo-oligomers.***A*, Box plot of homotypic GALLEX data for the core region of the wildtype Igα TMD (wt) alongside scanning mutagenesis results for point mutants along the length of the TMD, revealing that the E–X_10_–P motif dominates helix–helix interactions. All data are compared to the positive and negative controls, GpA and GpA G_83_I, respectively, and are provided in Miller units after normalization to expression level and the value obtained from G_83_I. Reported values are derived from three to six biological repeats. Asterisks denote mutants that are significantly different from wildtype with *p* < 0.05 (∗) or *p* < 0.01 (∗∗). *B*, Molecular model of Igα TMD homodimer obtained using CHI (see Experimental procedures), illustrating a putative TMD dimer stabilized by interactions involving residues E_148_ and P_159_ (shown as *spheres*). The helix–helix interaction interface in this model also contains residues I_144_, I_151_, C_155_, L_163_, and R_166_ (shown as *sticks*). *C*, Molecular models of the Igα TMD homodimer obtained using PREDDIMER. The two top-ranked (*i.e.*, highest F_SCOR_, see Table S1) structures are overlaid to show that both contain E_148_ and P_159_ at the helix–helix interface. CHI, CNS searching of helix interactions; GpA, glycophorin A; TMD, transmembrane domain.

Scanning mutagenesis was used to elucidate which amino acids within the TMD of Igα stabilize the strong self-association observed. Each amino acid from T_146_–L_163_ was substituted with either Ala or Ile (depending on the hydrophobicity of the native residue), and the resulting chimeras were investigated using the homotypic GALLEX assay (Fig. 4*A*). One-way ANOVA was used to reveal a significant difference in β-gal activity, and thus in association rates, between Igα mutants and the wildtype oligomer (F = 195.527, *p* < 0.001). Least significant difference *post hoc* testing was also used to identify specific Igα mutants significantly different to wildtype. Nine of the eighteen point mutants tested had no significant impact on the strength of the interaction within error. The remaining nine mutants yielded β-Gal activities significantly different from the wildtype Igα with either a *p* < 0.05 (∗) or *p* < 0.01 (∗∗) *versus* wildtype. As is clear from Figure 4*A*, only two mutations, E_148_I and P_159_I, yielded severe disruption of Igα TMD interactions. Computational models of human Igα TMD homodimers were produced to illustrate configurations that support these data using either the program CHI (CNS searching of helix interactions (46) or the PREDDIMER online prediction tool (47) as described in the Experimental procedures. Figure 3*B* shows a representative structural model returned from CHI searches in which E_148_ and P_159_ pack at the helix–helix interface along with I_151_, C_155_, and L_163_. To validate this model and better explore the ensemble of possible Igα homodimer configurations, a more recently developed tool called PREDDIMER was used (see Table S1 for parameters of all predicted models). An overlay of the two top-ranked Igα homodimer structures obtained from PREDDIMER is shown in Figure 4*C*, where E_148_ and P_159_ residues (*spheres*) are localized at or very near the helix–helix interface in both models. Two further dimer structures were also predicted, both of which had large tilt angles (Table S1), involved packing of an Ala and a Leu residue at the center of the TMD, and thus did not agree with the mutagenesis data. The GALLEX results in combination with computational data suggest that the Igα TMD can form stable homo-oligomers *via* an E–X_10_–P motif previously reported to mediate ER retention and interactions with the mIg homodimer (12).

### Interaction between the human Igα and Igβ transmembrane domains in detergent micelles

Interactions between the Igα and Igβ TMDs were investigated *in vitro via* chemical cross-linking and affinity chromatography with fluorescence detection. For both approaches, the His_6_-Igβ peptide was ideal as its larger mass made it resolvable from the Igα peptide on SDS-PAGE, its lack of fluorophore meant that Igα could be selectively monitored using fluorescence, and its His_6_ tag could be exploited for immobilized metal affinity chromatography (IMAC). Figure 2*C* shows the results from glutaraldehyde cross-linking of a constant concentration of the His_6_-Igβ peptide in the presence of increasing molar ratios of the Igα peptide. In the first lane (containing no Igα), the monomeric and dimeric His_6_-Igβ species are observed. As Igα concentration was increased (*i.e.*, from left to right), the His_6_-Igβ dimer (ββ) decreased in concentration, while a new band (αβ) increased in concentration. This new band is not at the MW of either the Igα or the Igβ homodimers and is most likely due to formation of an Igα–Igβ heterodimer.

IMAC was also used to investigate interactions between the Igα and Igβ TMD peptides. The Igα TMD peptide was loaded onto two separate IMAC columns containing (a) Ni-charged Sepharose resin and (b) Ni-charged Sepharose resin with bound His_6_-Igβ peptide. Elution of the Igα TMD peptide from each column was monitored using SDS-PAGE and fluorescence spectroscopy, as the Igα peptide has a maximum fluorescence emission wavelength at 347 nm (Fig. S2). The free Igα peptide eluted very early from the column containing only Ni-charged Sepharose resin. Both fluorescence (Fig. 2*D*, *closed circles*) and SDS-PAGE (Fig. S3*A*) indicated the majority of the peptide eluted in the first three fractions, equivalent to three column bed volumes (bed volume = 500 μl). For the column containing Ni-bound His_6_-Igβ peptide, where His_6_-Igβ binding was confirmed using SDS-PAGE (Fig. S3*B*), free Igα peptide was also detected in early fractions (Fig. 2*D* *open circles* and inset). However, addition of imidazole to the column released additional Igα peptide as well as the His_6_-Igβ peptide (see Fig. 2*D* inset lanes 13–17). These results are in agreement with those from cross-linking and suggest that the Igα and Igβ TMD peptides form productive interactions *in vitro*.

### Interaction between human Igα and Igβ transmembrane domains in a natural membrane environment

**Figure 5:**
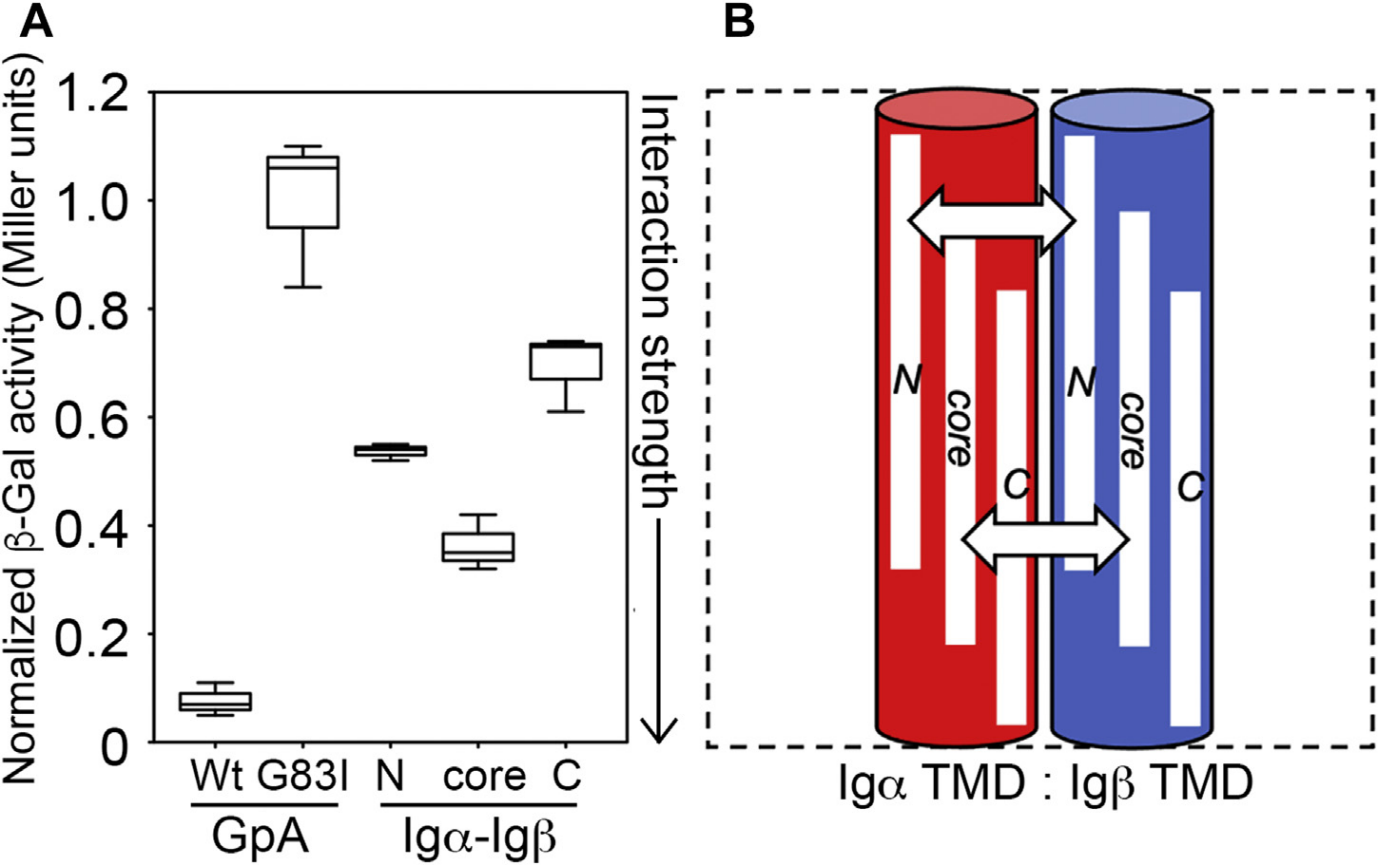
**Hetero-association of the BCR transmembrane domains (TMDs) in a natural membrane bilayer.***A*, Box plot of heterotypic GALLEX data for three TMD regions of wildtype Igα and Igβ (see Table 1 for sequences) screened for their ability to associate with one another. All data are compared to the positive and negative controls, GpA and GpA G_83_I, respectively, and are provided in Miller units after normalization to expression level and the value obtained from G_83_I. Reported values are derived from three to six biological repeats. *B*, Schematic summary of GALLEX data for the three different frames of the Igα and Igβ TMDs, with double-headed arrows indicating which regions of the TMDs associate most strongly. These data suggest that the Igα and Igβ TMDs contain sites of productive protein–protein interactions, with the strongest interactions observed in the core of the TMD and the weakest near the C-terminus. GpA, glycophorin A.

The GALLEX assay was utilized to investigate the degree to which Igα and Igβ TMDs hetero-oligomerize in a natural membrane bilayer. Figure 5*A* shows the GALLEX results for interactions between the N-terminal, core, and C-terminal regions of the human Igα and Igβ TMDs. Measurements were made for Igα and Igβ TMD regions located at similar positions within each TMD (*i.e.*, Igα core–Igβ core) and which would be expected to exist at similar depths in the membrane to approximate interactions that would take place between the full-length TMDs in their native environment of the human B-cell membrane. These results are summarized in a schematic in Figure 5*B*, with arrows between regions that yielded strong interactions relative to the positive control. All three TMD regions yielded interactions stronger than that of the negative control and clearly indicate that the Igα and Igβ TMDs (in isolation) associate with one another to some degree across much of their length, with the strongest interactions observed at the core of the TMDs and the weakest interactions toward the C-terminal portion of the TMDs. To our knowledge, these results are the first of their kind and demonstrate that the Igα and Igβ TMDs are sites of moderate to strong interactions that may stabilize the full-length Igα/β heterodimer *in vivo*.

### Heterotypic interaction is muticonfigurational but mediated in part by a central tryptophan

**Figure 6:**
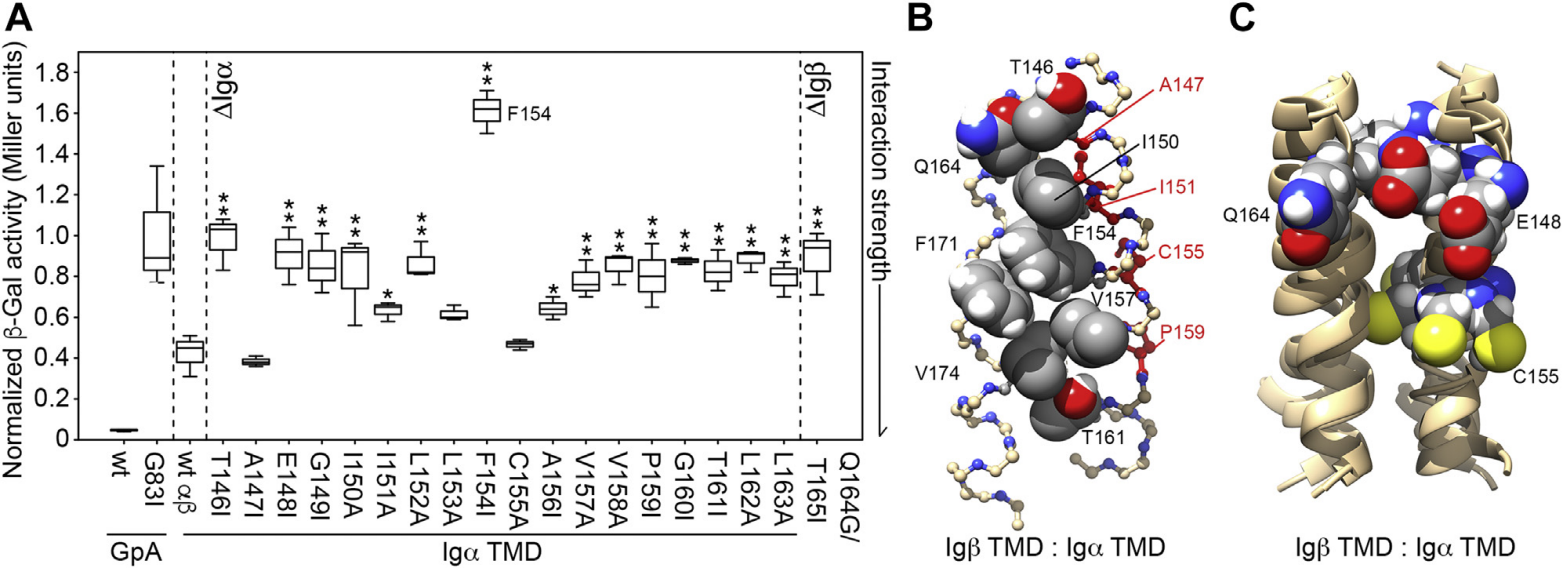
**Central tryptophan residue stabilizes interactions between Igα and Igβ TMDs.***A*, Box plot of heterotypic GALLEX data for the core region of wildtype human Igα and Igβ TMDs (wt αβ) alongside scanning mutagenesis results for point mutants along the length of the Igα TMD screened against the wildtype Igβ TMD, again compared to the positive and negative controls (GpA and GpA G_83_I) and normalized to expression level and average G_83_I value. Also shown are data for a double mutant of the Igβ TMD (Q_164_G T_165_I) screened against wildtype Igα TMD. *B*, Molecular model of the Igα–Igβ TMD interaction (produced using CHI) which best matched experimental data shown in panel (*A*), illustrating a putative TMD heterodimer stabilized by interactions involving F_154_ in Igα and F_171_ in Igβ in an orientation reflecting π–π stacking of the Phe side chains. In this model, T_146_, I_150_, F_154_, V_157_, and T_161_ in Igα pack against Q_164_, F_171_, and V_174_ in Igβ. Additionally, the interaction site in this model excludes A_147_, I_151_, C_155_, and P_159_ (shown in *red*). *C*, Molecular models of Igα–Igβ TMD heterodimer obtained using PREDDIMER. The four structures with F_SCOR_ values >2.0 (see Table S1) are overlaid to show the degree of configurational variation, with E_148_ and C_155_ in Igα and Q_164_ in Igβ shown as *spheres* as points of reference. CHI, CNS searching of helix interactions; GpA, glycophorin A; TMD, transmembrane domain.

Identification of specific residues that stabilized Igα–Igβ TMD interactions was carried out *via* heterotypic GALLEX screening of the wildtype Igβ core TMD region against the library of Igα point mutants described in Figure 4. The results of this screen are shown in Figure 6*A*, alongside the results from the positive and negative controls and the wildtype Igα TMD. While some periodicity can be seen across the data, most of the Igα substitutions tested were disruptive to Igα–Igβ TMD interactions: 13 of the 18 point mutants yielded β-Gal activities significantly different from wildtype, with a *p*-value < 0.01 (∗∗). The most disruptive mutant was F_154_I, suggesting that F_154_ is an important site of interaction. Apart from this residue, it was challenging to pinpoint from the GALLEX data a discrete interaction site/face in the Igα TMD that stabilized binding to Igβ. For this reason, we looked at which residues in Igα were likely to be excluded from the Igα–Igβ interaction interface and observed that mutation of A_147_, L_153_, and C_155_ had no significant impact on the strength of the interaction within error. As well as mutation of Igα, we created a double mutant in the Igβ TMD, Q_164_G T_165_I, which we screened against the wildtype Igα TMD in GALLEX (Fig. 6*A*). This mutant eliminated the only two polar residues in the entire core region of the Igβ TMD, interrupted the Q-X_10_-P motif proposed previously to be analogous to the E–X_10_–P motif in Igα (12), and is shown here to significantly disrupt interactions between the Igα and Igβ TMDs.

**Figure 7:**
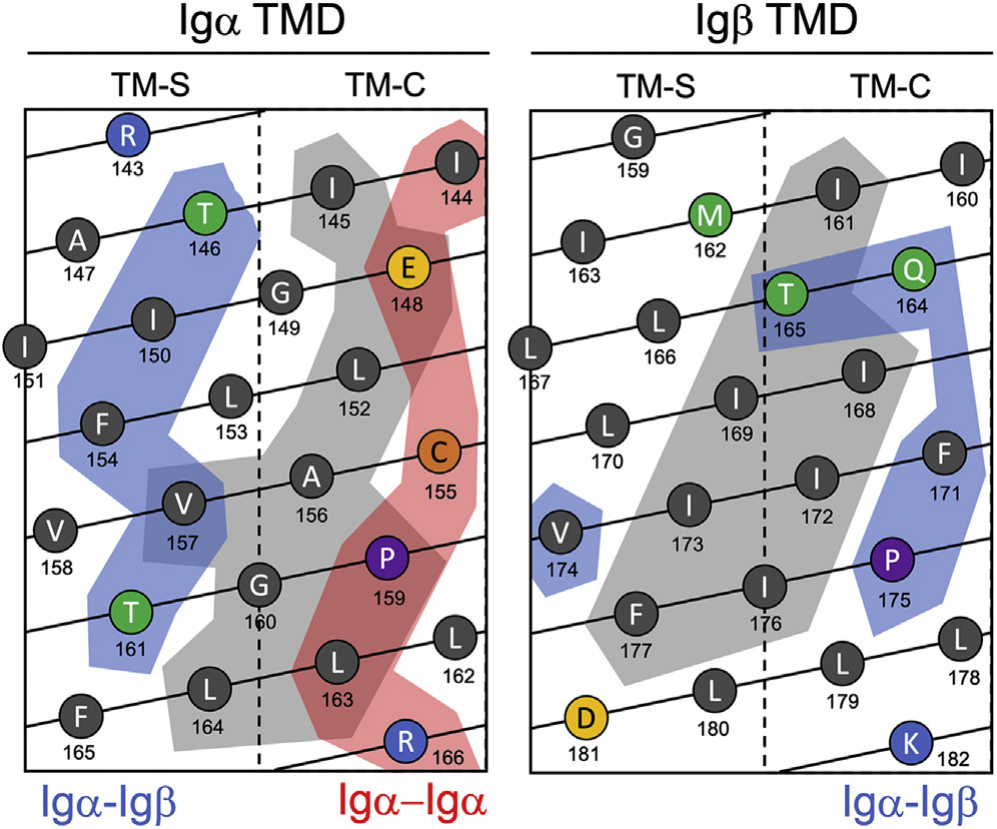
**Schematic summarizing two distinct interaction sites in the Igα TMD.** Helical net plots of the Igα and Igβ TMD residues, with the two evolutionarily conserved helical faces (TM-C and TM-S) shown. Residues proposed here to lie in the Igα homodimer interaction site are highlighted in *red*, and residues in both Igα and Igβ that lie in the heterodimer interaction site are highlighted in *blue*. For comparison, the residues proposed previously from molecular dynamics simulation (27) to lie in the heterodimer interaction site are highlighted in *gray*. TMD, transmembrane domain.

Computational models of the human Igα−Igβ TMD heterodimer produced using CHI and PREDDIMER are shown in Figure 6, *B* and *C*. CHI structures were examined to identify (a) those in which F_154_ formed part of the interaction site in Igα and (b) if it contained either Q_164_ or T_165_ at the interaction site in Igβ. Figure 6*B* shows the resulting heterodimer structural model returned from CHI. In this structure, Igα F_154_ packs against Igβ F_171_ in an arrangement that would promote π–π stacking of the Phe side chains. The helix–helix interaction site in Igα is composed of T_146_, I_150_, F_154_, V_157_, and T_161_, all of which disrupt wildtype interaction significantly (*p* < 0.01) when mutated. The helix–helix interaction site in Igβ includes V_174_, F_171_, and Q_164_, thus explaining the disruptive effect of the Q_164_G T_165_I mutant in Igβ. Finally, the model shown in Figure 6*B* excludes A_147_, I_151_, and C_155_ from the Igα–Igβ interaction site (see residues shown in *red*). These three residues lie on the same helical face which they share with P_159_, and comparison of the residues on this “excluded” face is in good agreement with those previously identified as the site of Igα–Igα interactions (Fig. 4*B*). While this model supports the mutagenesis data, it does not reflect the lack of an obvious and discrete interaction site in the Igα TMD. To explore the plasticity of Igα–Igβ heterodimer configurations, PREDDIMER was used. An overlay of the four top-ranked Igα–Igβ heterodimer structures (with F_SCOR_ values >2.0, see Table S1) from PREDDIMER is shown in Figure 6*C*. For a simple point of comparison, Q_164_ in the Igβ TMD and E_148_ and C_155_ in the Igα TMD are shown as *spheres*. In all models, the Q_164_ residue in the Igβ TMD lies at or near the helix–helix interface, suggesting high favorability of this arrangement. Conversely, the Igα TMD samples a wide range of configurations in these models as is apparent from the range of E_148_ and C_155_ positions shown in Figure 6*C*. These data suggest that the Igα–Igβ interaction is multiconfigurational with several minima possible, one of which is characterized by two distinct interactions sites: one that stabilizes Igα homodimeric (self) interactions and one that directs interactions with the Igβ TMD (summarized in Fig. 7).

## Discussion

The Igα and Igβ proteins make up the signaling component of the human BCR, a receptor that is critical to human immune response and plays a role in a range of clinically significant processes from vaccine response (1) to lymphoma (6). These proteins direct functional interactions with (a) one another, (b) the mIg, and (c) possibly other intact BCRs. Along with disulfide bridges located in the extracellular regions of the proteins, a growing body of work suggests that the TMDs of these proteins are important sites of interaction (9, 12, 21, 27, 48). Conversely, while the cytoplasmic domains of both Igα and Igβ are required for AP2-mediated endocytosis of antigen-linked BCR (15, 16), there is no evidence that the Igαβ heterodimer is itself stabilized by its cytoplasmic domains. The TMDs of Igα, Igβ, and mIg are thought to contain two evolutionarily conserved helical faces: one conserved between isotypes (TM-C) and one specific for each isotype (TM-S) (9, 12, 49). These regions are shown in Figure 7 for the Igα and Igβ TMDs. In this work, we have characterized the structure and homotypic/heterotypic interactions of the Igα and Igβ TMDs in isolation to experimentally determine if these regions can support protein–protein interactions and thus contribute more widely to functional assembly and activation of the BCR.

While a signaling-competent Igα/α BCR has yet to be described, our results nonetheless clearly demonstrate that the Igα TMD has a strong propensity to self-associate into homodimers *via* a highly conserved E–X_10_–P motif on its TM-C helical face (see region shaded in *red*, Fig. 7). Mutation of either residue in this motif leads to strong disruption of TMD self-association, whereas mutation of a central Cys residue on the same face (C_155_) had no significant impact, demonstrating that the interaction was not controlled by disulfide bonding (Fig. 4). While the widely accepted oligomeric state of Igα in the ER is that of a monomer (dimers have not been reported to date), the E–X_10_–P motif has been previously reported to function as an ER retention motif for unpaired Igα *in vivo* (12). Our new data would support a putative model in which Igα homodimer formation plays a key role in ER retention of this protein and, taken along with the previous mutagenesis data *in vivo* reported by Gottwick and coworkers (12), suggests disruption of Igα dimers leads to release of monomeric Igα to the cell’s surface. Given that such a monomer–dimer equilibrium is known to occur in Igβ, we propose that a similar mechanism may be used to control sorting of Igα. If this is the case, then retention of homodimeric Igα in the ER must represent an advantage to the cell. It has previously been shown that, on cross-linking, BCRs containing two Igα cytoplasmic domains are endocytosed more efficiently than the Igα/β wildtype BCR (15). Possibly this "hyperactive" form of BCR is disruptive to immune signaling, providing a motivation for retention of Igα homodimers in the ER and export only of Igα/β BCRs to the cell membrane. Alternatively, Igα TMD self-association may provide a driving force for assembly of higher-order BCR nanoclusters during BCR activation and signaling. Such an interaction is relevant in the context of either the cross-linking model or the dissociation activation model as it could provide a binding interface between individual BCRs (14). Indeed, it has been demonstrated in S2 *Drosophila* Schneider cells that when BCRs oligomerize, neighboring Igα chains are brought into close proximity (14). It has also been demonstrated that the endocytosis mediator AP2 interacts differentially with the cytoplasmic domain of Igα in the presence and absence of Igβ, preferentially binding to the membrane proximal endocytosis motif of Igβ when present and with that of Igα when Igβ was absent (16). As these domains were grafted onto the extracellular domains and TMDs of major histocompatibility complex-II, homo-oligomers were excluded from the study; however, our identification of a selective and stable Igα homodimer suggests a plausible interaction between AP2 and an Igα cytoplasmic homodimer, which might shed further light on the mechanism of endocytosis of either ligand-bound or signaling-aberrant (*i.e.*, Igα/α) BCRs.

There is strong evidence in the literature that excess Igβ forms a disulfide-linked homodimer (17). The biological significance of this is unclear, and the existence of mIg–Igβ/β BCRs is not reported in the literature (13, 50, 51), but Igβ TMD homo-oligomerization was investigated here. We observed weak self-association in the TMD of Igβ both *in vitro* and in a natural membrane (Figs. 2 and 3). This is in agreement with data published previously suggesting that the TMD of Igβ does not contribute significantly to formation of the Igβ homodimer. Instead, Igβ homodimers are predominantly stabilized by a disulfide bond in the extracellular Ig domain. Gottwick *et al.* showed clearly that mutation of Cys_135_ to Ser leads to a significant reduction in surface expression of Igβ homodimers (12). Conversely, mutation of the conserved Q–X_10_–P motif in the TMD of Igβ had variable effects on surface expression, with QP/AA mutants migrating to the surface in a similar manner to wildtype, while QP/KA mutants were retained in the ER. The Q–X_10_–P motif was thus interpreted by the authors as an ER retention signal for unpaired Igβ and not a requirement for Igβ homodimer formation, and our work supports these conclusions.

With respect to the role of TMD interactions in formation of the Igα/β heterodimer, we observed strong interactions between the TMDs of each protein both *in vitro* and *in vivo* (see Figs. 2 and 5). These results are the first of their kind and are at variance with the conclusions of Gottwick *et al.* (12) who proposed a model of BCR assembly in which the TMDs are dispensable for Igαβ heterodimerization and instead mediate interactions between mIg and the individual Igα and Igβ subunits. However, in this study only the impact of mutation of the E/Q–X_10_–P motifs within each TMD was investigated. Our mutageneis data suggest that the Igα/β heterodimer is multiconfigurational with respect to the Igα TMD (Fig. 6*C*), not exclusively involving the E–X_10_–P motif (which we suggest directs Igα homodimer formation), but more well defined with respect to Igβ and specifically the Gln residue from the Q–X_10_–P motif. We also note that, in the previous study, the Gln residue was mutated to Lys and the subsequent impact on cell surface expression of the Igα/β heterodimer evaluated. Such a Lys substitution at this position would be well tolerated in the TMD heterodimer we propose in Figure 6*B*, as it would still be able to form productive hydrogen bonding interactions with T_146_ in Igα (the Thr side chain can act as both an H-bond acceptor and an H-bond donor) and would explain why no impact on heterodimer formation was observed previously. Therefore, we propose that the TMDs of Igα and Igβ are sites of strong interactions that likely work in concert with other stabilizing interactions (such as extracellular disulfide bond formation) to guide assembly of the functional heterodimer.

This is in broad agreement with recent results from *in silico* molecular dynamics simulation (27) which indicated that the Igα and Igβ TMDs and juxtamembrane domains form stable interactions in model 1-palmitoyl-2-oleoyl-sn-glycero-3-phosphocholine bilayers. The TMD residues in their heterodimer models differ from the ones shown in Figure 6*B* here, and the results are compared in Figure 7. Briefly, we have proposed that a putative TMD heterodimer forms between residues on the TM-S face of Igα and the TM-C face of Igβ (shaded in *blue*), stabilized by π–π stacking between the side chains of well-conserved Phe residues located at the center of each TMD and polar interactions involving Thr and Gln residues near the N-terminus of each TMD. Friess *et al.* (27) proposed an interaction site that lies intermediate between the TM-C and TM-S faces in both proteins (shaded in *gray*) and involves the E–X_10_–P motif in Igα. These differences likely point to the plasticity of the Igα/β TMD interactions that is evident in our mutagenesis data (Fig. 6*A*) and highlights the need for more work to experimentally validate the form of this heterodimer. Nevertheless, our results add to building evidence implicating a role for the Igα and Igβ TMDs in heterodimer formation that may facilitate selection of the optimal configuration to direct (a) extracellular disulfide bond formation, (b) interactions with mIg, (c) signaling of the intact BCR, or (d) endocytic activity of the Igαβ cytoplasmic tails.

In summary, we have demonstrated here that an E–X_10_–P motif in the TMD of Igα drives formation of a strong Igα homodimer and the TMD of Igβ can also weakly self-associate. We have shown that the TMD of Igα strongly interacts with the TMD of Igβ but this interaction may be multiconfigurational, with one mode of interaction involving helical face of Igα that is completely distinct from that of the Igα homodimer and which contains highly conserved Thr and Phe residues. The Igα/α and Igα/β interactions we report here may therefore take place concurrently, allowing BCR oligomers to nucleate in a multivalent manner, consistent with either the cross-linking or the dissociation activation model of BCR signaling.

## Experimental procedures

### Peptide synthesis and purification

The TMDs of Igα and Igβ were predicted *via* analysis of the human Igα (UniProt ID CD79A_HUMAN) and Igβ (UniProt ID CD79B_HUMAN) sequences using Phobius. Synthetic peptides derived from the Igα TMD (residues 143–166) and the Igβ TMD (residues 158–181) were prepared with either non-native Trp residues or an N-terminal hexahistidine tag using F-moc chemistry and purified to 95% purity at Insight Biotechnology Limited. All peptide sequences are given in Table 1. Peptide purity was confirmed by matrix-assisted time of flight mass spectrometry (MALDI-TOF–MS, Bruker, see Figs. S4–S6) before subsequent lyophilization. The peptides were stored as dry powders at −20 °C until use.

### Circular dichroism spectroscopy

CD spectra were collected on a Jasco J-1500 spectropolarimeter (Jasco) equipped with Peltier temperature control and xenon light sources. Samples contained 80 μM peptide solubilized in 25 mM sodium phosphate (pH 7.4) containing 100 mM DPC. Spectra comprising eight averaged accumulations were recorded between 190 nm and 260 nm, with a bandwidth of 2 nm and a data pitch of 0.2 nm. The temperature inside the cell holder was maintained at 37 °C. The CD spectrum of the buffer was recorded as a blank and was subtracted from each protein spectrum.

### Fluorescence spectroscopy

The intrinsic fluorescence of the tryptophan residue added to the Igα peptide was monitored in IMAC fractions by fluorescence spectroscopy. Fluorescence emission spectra were acquired between 285 and 400 nm on a Jasco FP-6500 (Jasco) spectrofluorometer, equipped with a Jasco ADP-303T temperature controller, using an excitation wavelength of 295 nm, bandwidth of 3 nm, data pitch of 0.2 nm, and scanning speed of 200 nm/min. Measurements were collected at 25 °C.

### Immobilized metal affinity chromatography

Chelating Sepharose Fast Flow (Amersham Biosciences) was charged with Ni ions and washed extensively in 25 mM sodium phosphate buffer (pH 7.4) containing 50 mM DPC. To one half of the resin was added a solution of 100 uM His_6_-Igβ peptide solubilized in the same buffer conditions. The peptide and resin were mixed on a rotary mixer for 2 h at room temperature before pouring into a gravity flow column. An equivalent column, containing no added His_6_-Igβ peptide, was also prepared. Both columns had a bed volume of 500 μl. A 1-ml solution containing 100 uM Igα peptide solubilized in 25 mM sodium phosphate buffer (pH 7.4, 50 mM DPC) was prepared, and 500 μl of this solution was added to each column. Fractions of 500 μl were collected and analyzed by fluorescence spectroscopy and SDS-PAGE.

### Chemical cross-linking of synthetic peptides

Cross-linking reactions were carried out for 40 μM solutions of peptides dissolved in 25 mM sodium phosphate buffer, pH 7.4, containing 50 mM DPC. Eighteen millimolar glutaraldehyde (Sigma–Aldrich) was used to cross-link the peptides in solution *via* primary amine groups. The cross-linking reaction was terminated after either 10 or 30 min by the addition of 50 mM Tris-HCl, pH 8.

### SDS-PAGE

Cross-linking and IMAC samples were analyzed by SDS-PAGE using 16% Novex Tricine gels (Invitrogen) and Tricine running buffer (Invitrogen) and visualized by staining with silver nitrate (Sigma–Aldrich). Peptide migration was referenced to the prestained protein standard SeeBlue Plus2 (Invitrogen).

### Molecular modeling of transmembrane domain interactions

Computational analyses of Igα and Igβ TMD homodimers and heterodimers were performed using two different methods. First, the CNS searching of helix interactions (CHI) program, the details of which have been described previously (46, 52), was utilized on an 8-node dual 2.66-GHz Xenon processor Linux cluster (Streamline Computing). Starting structures incorporated both right-handed and left-handed crossing angles and an axis-to-axis distance between the helices of 10.4 Å. In a search of dimer interactions, the two helices were independently rotated about their central axis in 30° increments from 0 to 360°. After each rotation, molecular dynamics simulations were performed using simulated annealing of atomic coordinates. Four different molecular dynamics simulations were performed for each starting geometry, and energy minimization of structures was carried out before and after simulation. Groups of structures with a backbone root mean squared deviation of ≤1 Å were placed in clusters of 10 or more members, followed by calculation of an average structure for each cluster and energy minimization. The PREDDIMER prediction tool, a surface-based algorithm for prediction of dimer conformations, was also used (47). PREDDIMER utilizes the molecular hydrophobicity potential approach to map hydrophobic and hydrophilic properties onto helical surfaces and determine complementarity. The output is a set of structures ranked by quality of packing (F_SCOR_). Molecular graphics and analyses were performed using UCSF Chimera, developed by the Resource for Biocomputing, Visualization, and Informatics at the University of California, with support from NIH P41-GM103311 (53).

### The GALLEX assay

Homo- and hetero-association of the Igα and Igβ TMDs were studied in the *E. coli* inner membrane using the GALLEX assay, the details of which have been described previously (35). All plasmids and strains used were kindly provided by Prof. D. Schneider. DNA encoding an 18-residue portion of the TMD of interest (see Table 1 for all sequences) was ligated into the pBLM100 plasmid (for homotypic measurements) or the pALM100 plasmid (for heterotypic measurements) to yield a fusion protein containing the target TMD inserted between periplasmic-localizing maltose-binding protein (MalE) and the N-terminal DNA-binding domain of LexA (pBLM) or a mutant LexA' (pALM). GALLEX fusion proteins were also generated encoding the strongly dimeric TMD of GpA as a positive control and its weakly dimerizing point mutant G_83_I as a negative control. The GALLEX fusion proteins were then transfected by electroporation and expressed in *E. coli* strain SU101 in the presence of 100 μg/ml ampicillin (homotypic measurements) or in *E. coli* strain SU202 in the presence of 50 μg/ml ampicillin and 5 μg/ml tetracycline (heterotypic measurements) after induction with 10 μM IPTG at 37 °C. In all cases, TMD interactions lead to repression of β-gal expression as observed using spectroscopic detection (specifically absorbance at 420 nm) of the breakdown of the substrate ortho-Nitrophenyl-β-galactoside to ortho-Nitrophenol. β-Gal activity was reported in Miller units and is inversely proportional to the strength of TMD interactions, as previously reported (35). Expression levels for all GALLEX constructs were quantified *via* Western blot analysis using antibodies against the MalE domain and subsequent quantification using the ImageJ software (National Institutes of Health) (54). Correct insertion and orientation of all chimeras in the *E. coli* inner membrane was confirmed using the MalE complementation assay, where NT326 MalE–deficient cells are grown on M9 agar plates containing 0.4% maltose or by protease sensitivity in a spheroplast assay (55). For all GALLEX data, a minimum of three biological repeats were measured and the median, interquartile range, and extremes of each dataset were reported in box plots. GALLEX mutagenesis data were analyzed with one-way ANOVA and least significant difference *post hoc* tests.

## Data availability

All data created during this research is openly available from the Warwick Research Archive Portal (WRAP) at https://wrap.warwick.ac.uk/164368.

## Supporting information

This article contains supporting information.

## Conflict of interest

The authors declare that they have no conflicts of interest with the contents of this article.
