# Supplementary material for: Characterization of interactions within the Igα/Igβ transmembrane domains of the human B-cell receptor provides insights into receptor assembly
Source: J Biol Chem. 2022 Mar 18;298(5):101843. doi: 10.1016/j.jbc.2022.101843 (PMC9018394; doi:10.1016/j.jbc.2022.101843)
Supplement: Supplemental Figures S1–S6 and Table S1 [file mmc1.docx]

***SUPPORTING INFORMATION for***

*Characterization of interactions within the Igα/Igβ transmembrane domains of the human B-cell receptor provides insights into receptor assembly*

Christine Lockey, Hannah Young, Jessica Brown and Ann M. Dixon^*^

**Running Title:** Interactions of Igα/β transmembrane domains

Department of Chemistry, University of Warwick, Coventry, CV4 7AL, UK.

* To whom correspondence should be addressed: Dr. Ann M. Dixon, Department of Chemistry, University of Warwick, Coventry, CV4 7AL, UK, Telephone: +44 2476 150037; FAX: +44 2476 524112; email: ann.dixon@warwick.ac.uk

**Table S1:** Parameters of helical dimers predicted using PREDDIMER algorithm

| **Dimer** | **Sequence** | **Crossing angle χ, deg** | **F_SCOR_** | **Rotn. angle α_1_, deg** | **Rotn. angle α_2_, deg** |
| --- | --- | --- | --- | --- | --- |
| Igα−α | RIITAEGIILLFCAVVPGTLLLFR | 25.2 | 2.29 | 158 | 158 |
|  |  | -6.8 | 2.28 | 104 | 110 |
|  |  | -55.1 | 1.71 | -52 | -52 |
|  |  | 60.0 | 1.65 | 206 | 248 |
| Igα−β | α-IITAEGIILLFCAVVPGTLLL  β-IIMIQTLLIILFIIVPIFLLL | 15.2 | 2.47 | 266 | 296 |
|  |  | -15.2 | 2.27 | -76 | -100 |
|  |  | 50.0 | 2.11 | 284 | 344 |
|  |  | -50.0 | 2.10 | -82 | -64 |

**
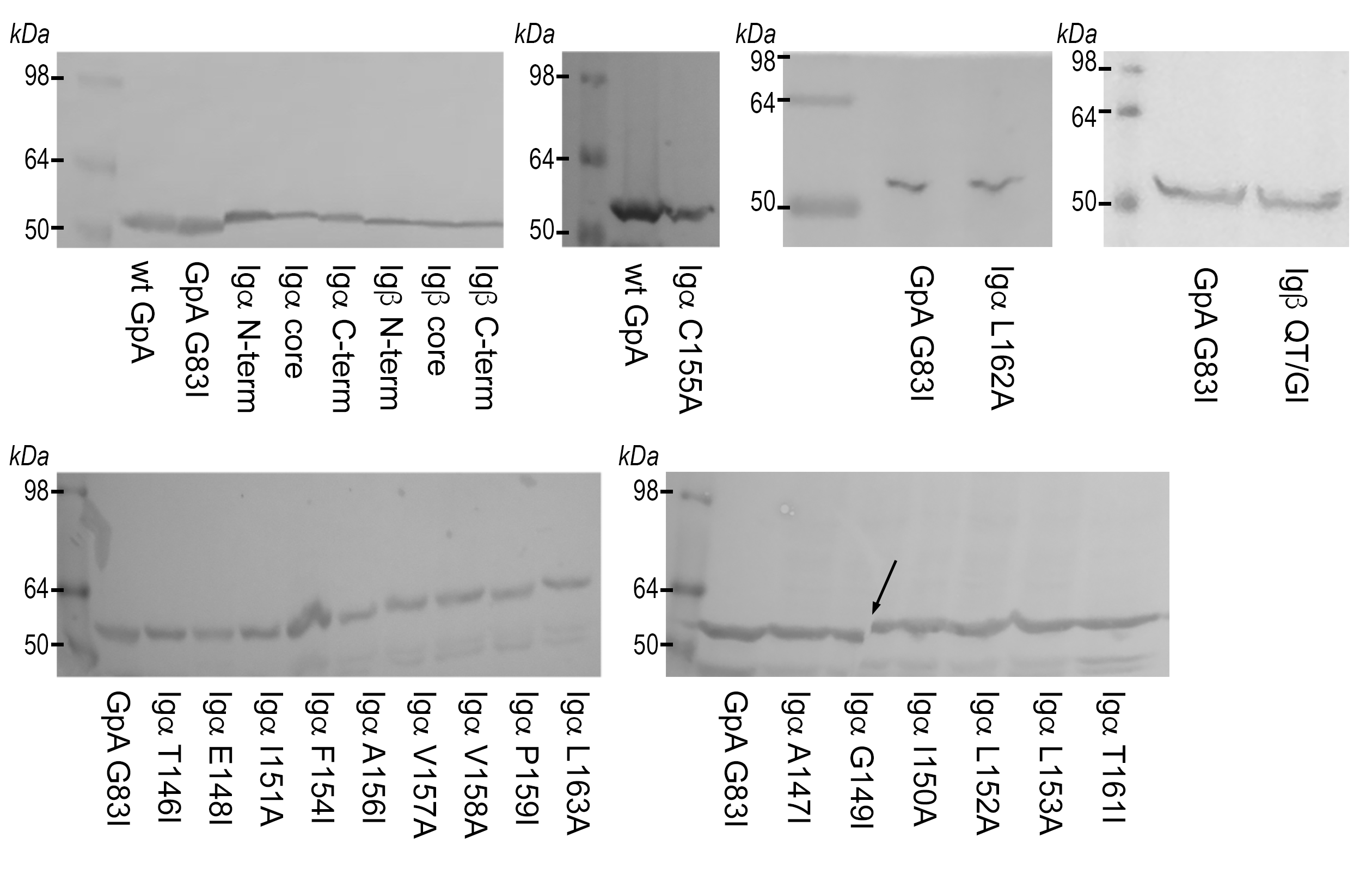
**

**Figure S1: Quantification of GALLEX chimerae expression levels by Western blot and analysis in ImageJ.** Cell cultures were normalized with respect to optical density at 600 nm. Blots were generated as described in Experimental Procedures. The intensity of each band was quantified using the image analysis software ImageJ, relative to the intensity of the negative control chimera GpA G_83_I. Protein migration was referenced to the prestained protein standard SeeBlue Plus2 (Invitrogen). Arrow in final panel indicates a small tear in the gel, distorting the resulting band.


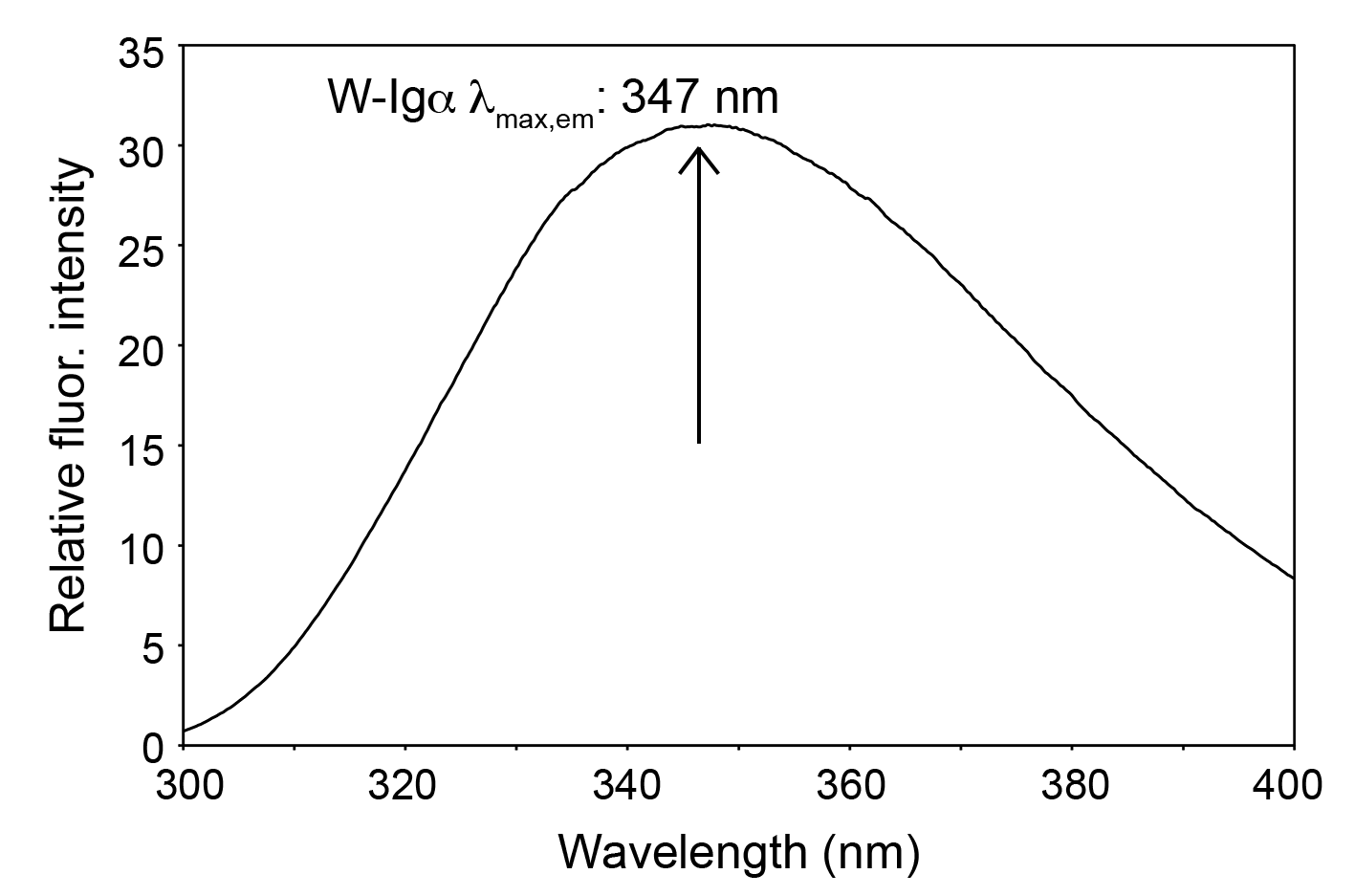


**Figure S2:** Fluorescence emission spectrum of the Igα TMD peptide using an excitation wavelength of 295 nm to excite the non-native tryptophan residue placed in the sequence.


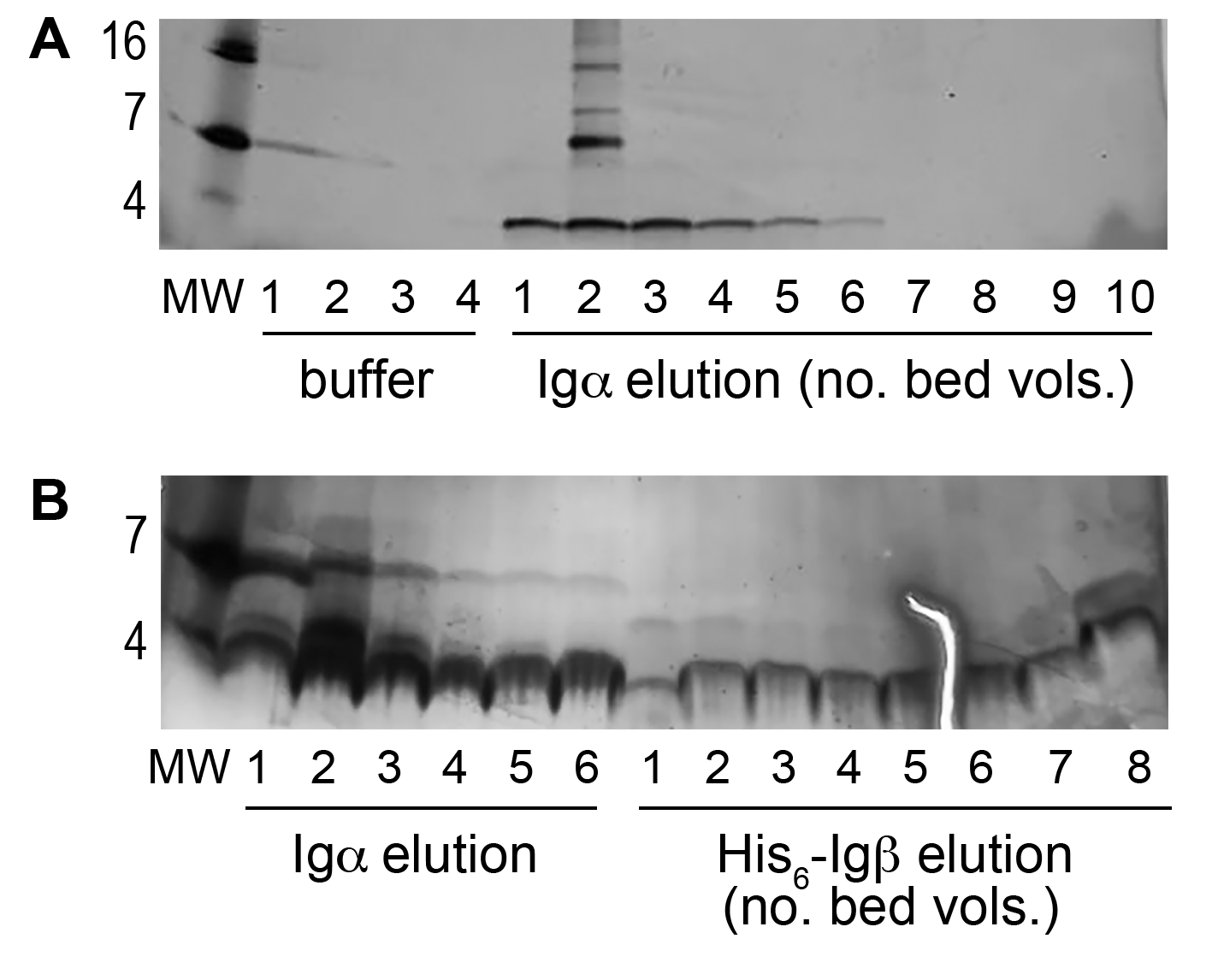


**Figure S3:** **SDS-PAGE analyses of IMAC elution fractions.** **(A)** Elution of the Igα TMD peptide from an IMAC column containing Ni-charged sepharose resin only. The column was washed with four bed volumes of buffer (1-4) before applying the peptide and collecting 10 x 500 μL fractions (1-10, one bed volume = 500 μL). The unbound Igα peptide eluted in the first six fractions, with the majority eluting in fraction 2. **(B)** SDS-PAGE was also used to monitor binding of the His_6_-Igβ peptide to Ni-charged sepharose resin. The peptide was mixed with the resin for two hours, the mixture was packed into a column, and the column was washed with buffer and fractions collected. These samples were loaded alongside Igα fractions 1-6 (from panel A) on the same gel to directly compare intensity of bands and thus judge if His_­6_-Igβ was bound to the resin. Silver staining of the gel shows that, compared with Igα, very little His_­6_-Igβ peptide was eluted from the column and therefore must be bound via its His_6_ tag. Peptides were visualized via staining with silver nitrate and the buffer used throughout all IMAC experiments was 25 mM sodium phosphate buffer (pH 7.4) containing 50 mM DPC detergent.

**
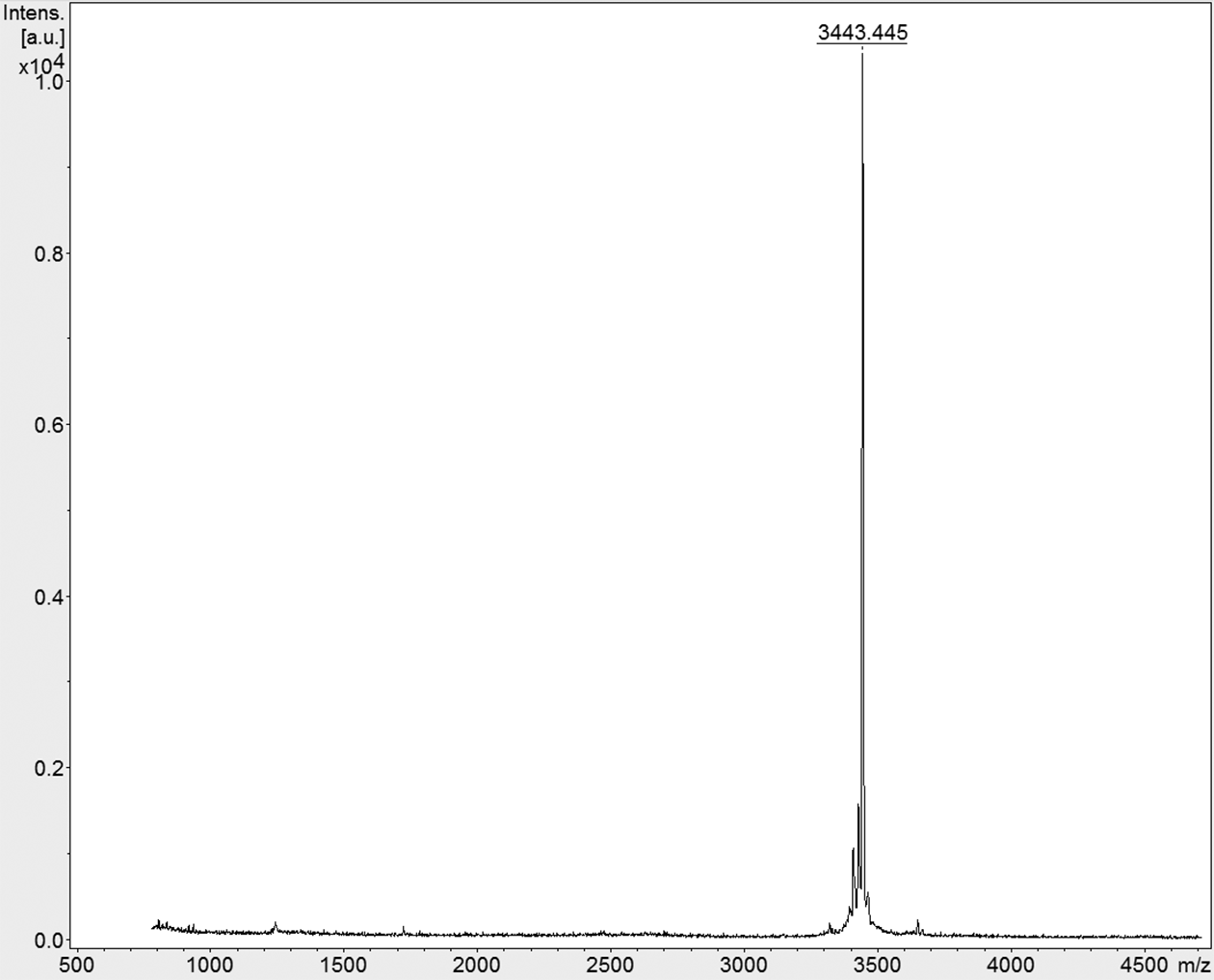
**

**Figure S4.** Matrix-assisted time of flight (MALDI-TOF) mass spectrum of the Igα TMD peptide used in this work (theoretical molecular weight = 3443 Da).

**
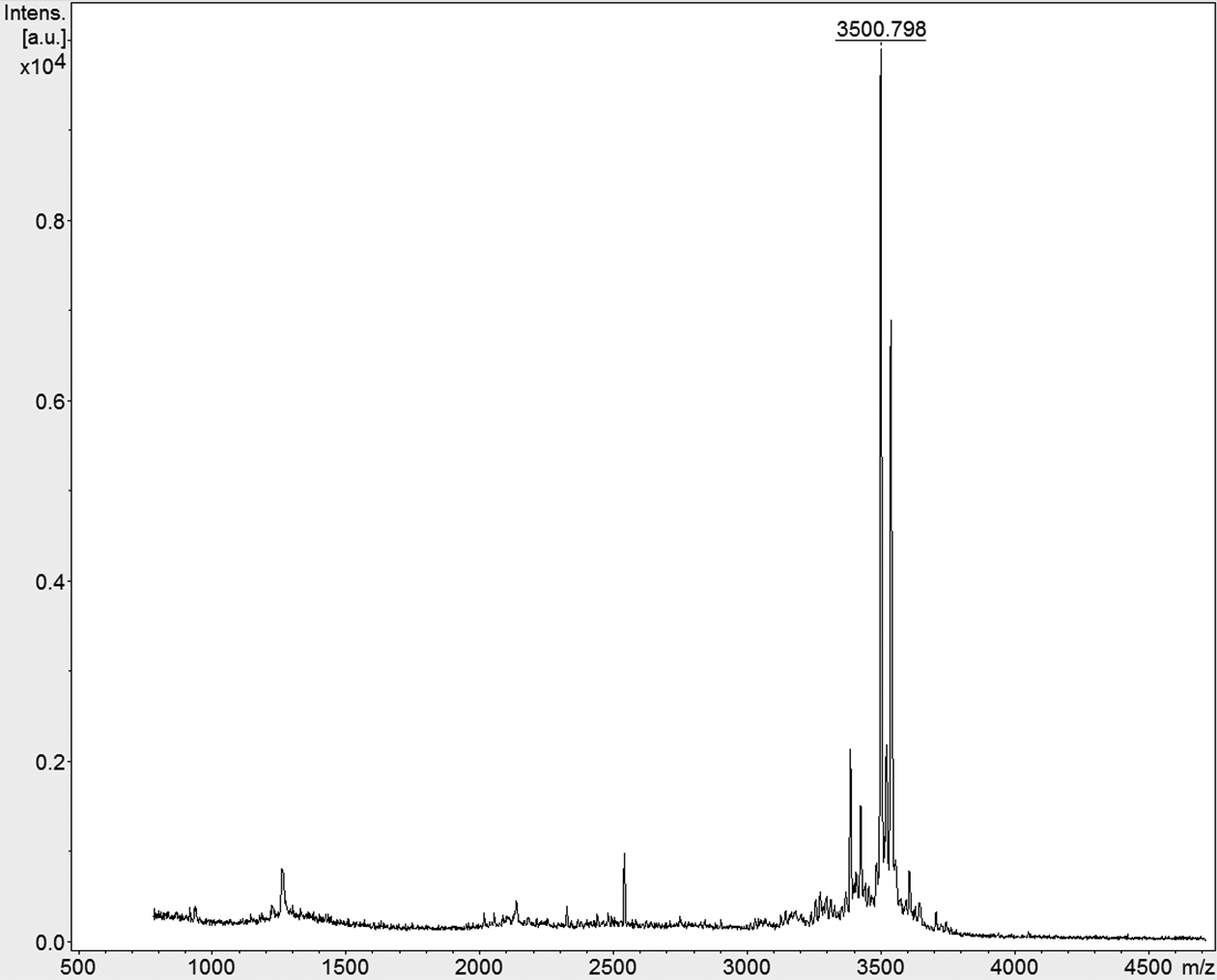
**

**Figure S5.** Matrix-assisted time of flight (MALDI-TOF) mass spectrum of the Igβ TMD peptide used in this work (theoretical molecular weight = 3500 Da).


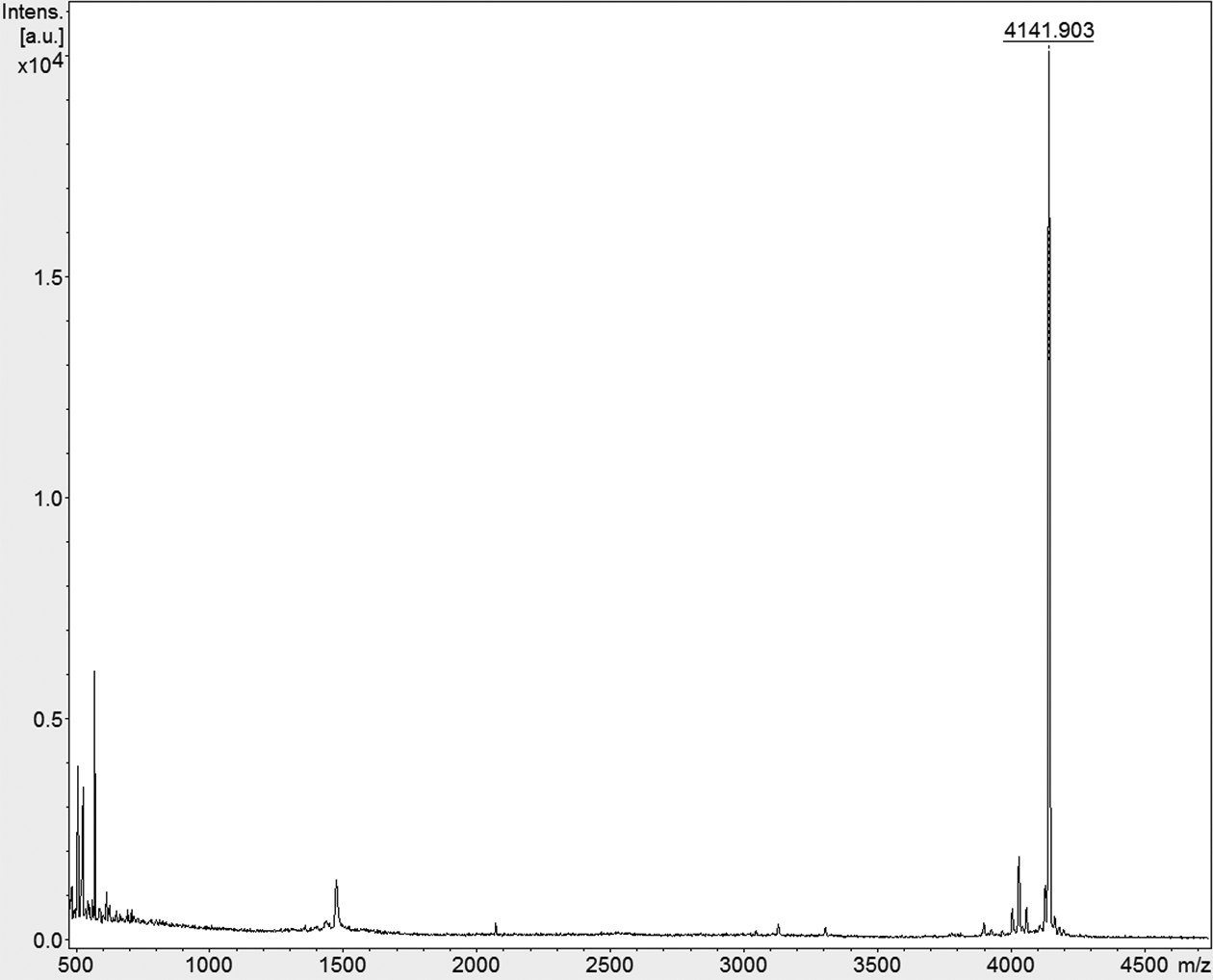


**Figure S6.** Matrix-assisted time of flight (MALDI-TOF) mass spectrum of the His_6_-Igβ TMD peptide used in this work (theoretical molecular weight = 4137 Da).
